# Ethyl 4-anilino-3-nitrobenzoate

**DOI:** 10.1107/S1600536808041329

**Published:** 2008-12-10

**Authors:** Hao-Yuan Li, Bo-Nian Liu, Shi-Gui Tang, Cheng Guo

**Affiliations:** a College of Science, Nanjing University of Technology, Xinmofan Road No. 5, Nanjing 210009, People’s Republic of China; b College of Life Sciences and Pharmaceutical Engineering, Nanjing University of Technology, Nanjing 210009, People’s Republic of China

## Abstract

In the title compound, C_15_H_14_N_2_O_4_, the aromatic rings are oriented at a dihedral angle of 78.33 (3)°. An intra­molecular N—H⋯O hydrogen bond results in a non-planar six-membered ring with a flattened-boat conformation. In the crystal structure, inter­molecular N—H⋯O hydrogen bonds link the mol­ecules. π–π contacts between the phenyl rings and both the phenyl and benzene rings, [centroid–centroid distances = 3.841 (3) and 3.961 (3) Å] may further stabilize the structure.

## Related literature

For bond-length data, see: Allen *et al.* (1987 ▶). For ring puckering parameters, see: Cremer & Pople (1975 ▶).
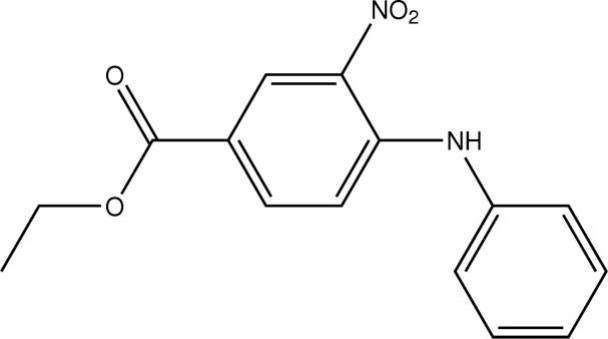


## Experimental

### 

#### Crystal data

C_15_H_14_N_2_O_4_
*M* *_r_* = 286.28
Monoclinic, 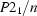

*a* = 10.683 (2) Å
*b* = 9.905 (2) Å
*c* = 13.698 (3) Å
β = 105.05 (3)°
*V* = 1399.7 (5) Å^3^
*Z* = 4
Mo *K*α radiation
μ = 0.10 mm^−1^
*T* = 294 (2) K
0.30 × 0.20 × 0.20 mm

#### Data collection

Enraf-Nonius CAD-4 diffractometer
Absorption correction: ψ scan (North *et al.*, 1968 ▶) *T*_min_ = 0.971, *T*_max_ = 0.980
2647 measured reflections
2508 independent reflections
1519 reflections with *I* > 2σ(*I*)
*R*_int_ = 0.051
3 standard reflections frequency: 120 min intensity decay: none

#### Refinement

*R*[*F* ^2^ > 2σ(*F* ^2^)] = 0.071
*wR*(*F* ^2^) = 0.199
*S* = 1.00
2508 reflections
190 parameters
H-atom parameters constrained
Δρ_max_ = 0.31 e Å^−3^
Δρ_min_ = −0.32 e Å^−3^

### 

Data collection: *CAD-4 Software* (Enraf–Nonius, 1989 ▶); cell refinement: *CAD-4 Software*; data reduction: *XCAD4* (Harms & Wocadlo, 1995 ▶); program(s) used to solve structure: *SHELXS97* (Sheldrick, 2008 ▶); program(s) used to refine structure: *SHELXL97* (Sheldrick, 2008 ▶); molecular graphics: *ORTEP-3 for Windows* (Farrugia, 1997 ▶); software used to prepare material for publication: *SHELXL97*.

## Supplementary Material

Crystal structure: contains datablocks global, I. DOI: 10.1107/S1600536808041329/hk2595sup1.cif

Structure factors: contains datablocks I. DOI: 10.1107/S1600536808041329/hk2595Isup2.hkl

Additional supplementary materials:  crystallographic information; 3D view; checkCIF report

## Figures and Tables

**Table 1:** Hydrogen-bond geometry (Å, °)

*D*—H⋯*A*	*D*—H	H⋯*A*	*D*⋯*A*	*D*—H⋯*A*
N2—H2*A*⋯O3	0.86	1.98	2.623 (5)	131
N2—H2*A*⋯O2^i^	0.86	2.31	2.978 (4)	134

Symmetry code: (i) 
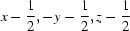
.

**Fig. 1.**
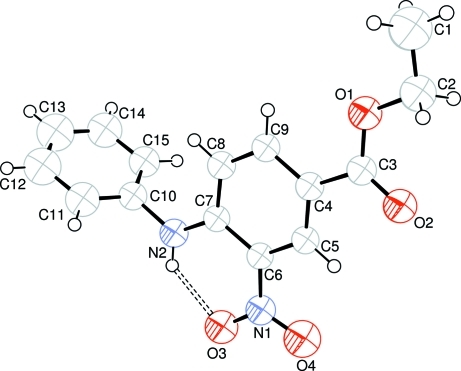
The molecular structure of the title molecule, with the atom-numbering scheme. Hydrogen bond is shown as dashed line.

**Fig. 2.**
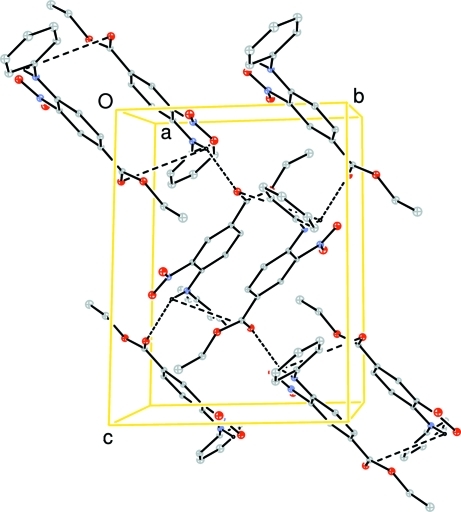
A partial packing diagram of the title compound. Hydrogen bonds are shown as dashed lines.

## supplementary crystallographic information

### Comment

Some derivatives of benzoic acid are important chemical materials. We report herein the crystal structure of the title compound.

In the molecule of the title compound (Fig 1), the bond lengths (Allen *et al.*,  1987) and angles are within normal ranges. Rings A (C4-C9) and B (C10-C15) are, of course, planar, and they are oriented at a dihedral angle of 78.33 (3)°. The intramolecular N-H···O hydrogen bond (Table 1) results in a nonplanar six-membered ring C (O3/N1/N2/C6/C7/H2A), having total puckering amplitude, Q_T_, of 0.131 (2) Å, flattened-boat conformation [φ = 140.37 (3)° and θ = 75.09 (4)°] (Cremer & Pople, 1975).

In the crystal structure, intermolecular N-H···O hydrogen bonds (Table 1) link the molecules (Fig. 2), in which they may be effective in the stabilization of the structure. The π-π contacts between the phenyl rings and the phenyl and the benzene rings, Cg1—Cg1^i^ and Cg1—Cg2^ii^ [symmetry codes: (i) 1 - x, 1 - y, 1 - z; (ii) x - 1/2, 1/2 - y, z - 1/2, where Cg1 and Cg2 are centroids of the rings A (C4-C6) and B (C10-C15), respectively] may further stabilize the structure, with centroid-centroid distances of 3.841 (3) Å and 3.961 (3) Å.

### Experimental

For the preparation of the title compound, ethyl 4-chloro-3-nitrobenzoate (5.0 g, 0.022 mol) was heated in fresh distilled aniline (10 ml) for 18 h at 393 K, and then ethanol (50 ml) was added, at room temperature. The yellow precipitate was sucked, washed with cold ethanol (2 X 20 ml), and then dried (yield; 4.7 g, 75%). Crystals suitable for X-ray analysis were obtained by slow evaporation of an ethanol solution.

### Refinement

H atoms were positioned geometrically, with N-H = 0.86 Å (for NH) and C-H = 0.93, 0.97 and 0.96 Å for aromatic, methylene and methyl H, respectively, and constrained to ride on their parent atoms, with U_iso_(H) = xU_eq_(C,N), where x = 1.5 for methyl H and x = 1.2 for all other H atoms.

### Crystal data

**Table:**

C_15_H_14_N_2_O_4_	*F*(000) = 600
*M**_r_* = 286.28	*D*_x_ = 1.358 Mg m^−^^3^
Monoclinic, *P*2_1_/*n*	Mo *K*α radiation, λ = 0.71073 Å
Hall symbol: -P 2yn	Cell parameters from 25 reflections
*a* = 10.683 (2) Å	θ = 10–13°
*b* = 9.905 (2) Å	µ = 0.10 mm^−^^1^
*c* = 13.698 (3) Å	*T* = 294 K
β = 105.05 (3)°	Block, colorless
*V* = 1399.7 (5) Å^3^	0.30 × 0.20 × 0.20 mm
*Z* = 4	

### Data collection

**Table:**

Enraf-Nonius CAD-4 diffractometer	1519 reflections with *I* > 2σ(*I*)
Radiation source: fine-focus sealed tube	*R*_int_ = 0.051
graphite	θ_max_ = 25.2°, θ_min_ = 2.2°
ω/2θ scans	*h* = −12→12
Absorption correction: ψ scan (North *et al.*, 1968)	*k* = 0→11
*T*_min_ = 0.971, *T*_max_ = 0.980	*l* = 0→16
2647 measured reflections	3 standard reflections every 120 min
2508 independent reflections	intensity decay: none

### Refinement

**Table:**

Refinement on *F*^2^	Primary atom site location: structure-invariant direct methods
Least-squares matrix: full	Secondary atom site location: difference Fourier map
*R*[*F*^2^ > 2σ(*F*^2^)] = 0.071	Hydrogen site location: inferred from neighbouring sites
*wR*(*F*^2^) = 0.199	H-atom parameters constrained
*S* = 1.00	*w* = 1/[σ^2^(*F*_o_^2^) + (0.050*P*)^2^ + 3.4*P*] where *P* = (*F*_o_^2^ + 2*F*_c_^2^)/3
2508 reflections	(Δ/σ)_max_ < 0.001
190 parameters	Δρ_max_ = 0.31 e Å^−^^3^
0 restraints	Δρ_min_ = −0.32 e Å^−^^3^

### Special details

**Table:**

Geometry. All e.s.d.'s (except the e.s.d. in the dihedral angle between two l.s. planes) are estimated using the full covariance matrix. The cell e.s.d.'s are taken into account individually in the estimation of e.s.d.'s in distances, angles and torsion angles; correlations between e.s.d.'s in cell parameters are only used when they are defined by crystal symmetry. An approximate (isotropic) treatment of cell e.s.d.'s is used for estimating e.s.d.'s involving l.s. planes.
Refinement. Refinement of *F*^2^ against ALL refle (Sheldrick, 2008)ctions. The weighted *R*-factor *wR* and goodness of fit *S* are based on *F*^2^, conventional *R*-factors *R* are based on *F*, with *F* set to zero for negative *F*^2^. The threshold expression of *F*^2^ > σ(*F*^2^) is used only for calculating *R*-factors(gt) *etc*. and is not relevant to the choice of reflections for refinement. *R*-factors based on *F*^2^ are statistically about twice as large as those based on *F*, and *R*- factors based on ALL data will be even larger.

### Fractional atomic coordinates and isotropic or equivalent isotropic displacement parameters (Å^2^)

**Table:**

	*x*	*y*	*z*	*U*_iso_*/*U*_eq_	
O1	0.0229 (3)	0.1219 (3)	0.2353 (2)	0.0602 (9)	
O2	0.1935 (2)	−0.0076 (3)	0.2291 (2)	0.0605 (8)	
O3	−0.0506 (3)	−0.4222 (3)	−0.1083 (3)	0.0705 (10)	
O4	0.1353 (3)	−0.3433 (4)	−0.0297 (3)	0.0852 (12)	
N1	0.0155 (3)	−0.3404 (4)	−0.0480 (3)	0.0560 (9)	
N2	−0.2640 (3)	−0.2968 (4)	−0.1003 (2)	0.0523 (9)	
H2A	−0.2321	−0.3625	−0.1271	0.063*	
C1	0.0290 (5)	0.2946 (6)	0.3560 (4)	0.0943 (19)	
H1A	0.0836	0.3471	0.4092	0.141*	
H1B	−0.0328	0.2455	0.3820	0.141*	
H1C	−0.0159	0.3535	0.3027	0.141*	
C2	0.1084 (4)	0.1999 (5)	0.3166 (4)	0.0683 (14)	
H2B	0.1541	0.1399	0.3700	0.082*	
H2C	0.1718	0.2486	0.2909	0.082*	
C3	0.0774 (4)	0.0183 (4)	0.2003 (3)	0.0489 (10)	
C4	−0.0131 (3)	−0.0606 (4)	0.1202 (3)	0.0464 (10)	
C5	0.0350 (3)	−0.1607 (4)	0.0723 (3)	0.0452 (10)	
H5A	0.1238	−0.1764	0.0897	0.054*	
C6	−0.0450 (3)	−0.2399 (4)	−0.0018 (3)	0.0434 (9)	
C7	−0.1821 (3)	−0.2191 (4)	−0.0295 (3)	0.0438 (9)	
C8	−0.2275 (3)	−0.1155 (4)	0.0203 (3)	0.0486 (10)	
H8A	−0.3161	−0.0984	0.0034	0.058*	
C9	−0.1479 (3)	−0.0370 (4)	0.0934 (3)	0.0465 (10)	
H9A	−0.1827	0.0313	0.1249	0.056*	
C10	−0.4030 (3)	−0.2732 (5)	−0.1322 (3)	0.0526 (11)	
C11	−0.4831 (4)	−0.3588 (6)	−0.1004 (4)	0.0754 (15)	
H11A	−0.4487	−0.4284	−0.0559	0.090*	
C12	−0.6166 (5)	−0.3428 (7)	−0.1342 (5)	0.0907 (19)	
H12A	−0.6722	−0.4012	−0.1127	0.109*	
C13	−0.6650 (4)	−0.2397 (7)	−0.1995 (5)	0.096 (2)	
H13A	−0.7543	−0.2280	−0.2219	0.115*	
C14	−0.5848 (4)	−0.1536 (6)	−0.2324 (4)	0.0854 (18)	
H14A	−0.6199	−0.0841	−0.2768	0.103*	
C15	−0.4501 (4)	−0.1691 (5)	−0.1999 (4)	0.0654 (13)	
H15B	−0.3944	−0.1122	−0.2226	0.078*	

### Atomic displacement parameters (Å^2^)

**Table:**

	*U*^11^	*U*^22^	*U*^33^	*U*^12^	*U*^13^	*U*^23^
O1	0.0423 (16)	0.0602 (19)	0.0576 (18)	−0.0073 (14)	−0.0236 (13)	−0.0105 (15)
O2	0.0306 (14)	0.075 (2)	0.0604 (18)	−0.0033 (14)	−0.0167 (12)	0.0023 (16)
O3	0.0482 (17)	0.067 (2)	0.080 (2)	0.0093 (16)	−0.0131 (16)	−0.0220 (18)
O4	0.0283 (15)	0.115 (3)	0.104 (3)	0.0123 (17)	0.0021 (16)	−0.016 (2)
N1	0.0367 (18)	0.069 (2)	0.056 (2)	0.0118 (18)	−0.0003 (16)	−0.0016 (19)
N2	0.0263 (16)	0.066 (2)	0.056 (2)	−0.0001 (15)	−0.0046 (14)	−0.0167 (18)
C1	0.090 (4)	0.092 (4)	0.082 (4)	−0.013 (3)	−0.013 (3)	−0.026 (3)
C2	0.057 (3)	0.068 (3)	0.061 (3)	−0.013 (2)	−0.018 (2)	−0.017 (3)
C3	0.040 (2)	0.053 (3)	0.042 (2)	−0.0083 (19)	−0.0115 (17)	0.009 (2)
C4	0.0278 (18)	0.052 (2)	0.045 (2)	−0.0041 (17)	−0.0165 (16)	0.0055 (19)
C5	0.0262 (18)	0.052 (2)	0.046 (2)	−0.0026 (17)	−0.0117 (16)	0.0101 (19)
C6	0.0299 (18)	0.053 (2)	0.041 (2)	0.0049 (17)	−0.0023 (16)	0.0030 (18)
C7	0.0240 (17)	0.049 (2)	0.048 (2)	−0.0009 (17)	−0.0090 (15)	−0.0001 (19)
C8	0.0233 (17)	0.060 (3)	0.053 (2)	0.0000 (17)	−0.0071 (16)	−0.001 (2)
C9	0.0327 (19)	0.052 (2)	0.047 (2)	−0.0006 (17)	−0.0021 (16)	−0.0051 (19)
C10	0.0226 (18)	0.068 (3)	0.057 (3)	−0.0039 (19)	−0.0082 (17)	−0.023 (2)
C11	0.047 (3)	0.107 (4)	0.072 (3)	−0.015 (3)	0.014 (2)	−0.016 (3)
C12	0.048 (3)	0.118 (5)	0.114 (5)	−0.026 (3)	0.036 (3)	−0.033 (4)
C13	0.024 (2)	0.127 (6)	0.122 (5)	−0.009 (3)	−0.006 (3)	−0.064 (5)
C14	0.039 (3)	0.098 (4)	0.097 (4)	0.016 (3)	−0.022 (3)	−0.030 (3)
C15	0.033 (2)	0.059 (3)	0.089 (3)	0.002 (2)	−0.013 (2)	−0.008 (3)

### Geometric parameters (Å, °)

**Table:**

O1—C2	1.463 (5)	C5—C6	1.387 (5)
O1—C3	1.329 (5)	C5—H5A	0.9300
O2—C3	1.227 (4)	C6—C7	1.429 (5)
N1—O3	1.239 (4)	C7—C8	1.387 (5)
N1—O4	1.240 (4)	C8—C9	1.374 (5)
N1—C6	1.422 (5)	C8—H8A	0.9300
N2—C7	1.363 (5)	C9—H9A	0.9300
N2—C10	1.454 (4)	C10—C11	1.354 (6)
N2—H2A	0.8600	C10—C15	1.390 (6)
C1—C2	1.458 (7)	C11—C12	1.390 (7)
C1—H1A	0.9600	C11—H11A	0.9300
C1—H1B	0.9600	C12—C13	1.367 (9)
C1—H1C	0.9600	C12—H12A	0.9300
C2—H2B	0.9700	C13—C14	1.366 (8)
C2—H2C	0.9700	C13—H13A	0.9300
C3—C4	1.483 (5)	C14—C15	1.400 (6)
C4—C5	1.360 (6)	C14—H14A	0.9300
C4—C9	1.410 (5)	C15—H15B	0.9300
			
C3—O1—C2	115.9 (3)	C5—C6—N1	117.2 (3)
O3—N1—O4	120.0 (4)	C5—C6—C7	120.5 (4)
O3—N1—C6	120.6 (3)	N2—C7—C8	121.7 (3)
O4—N1—C6	119.4 (4)	N2—C7—C6	122.1 (4)
C7—N2—C10	122.7 (3)	C8—C7—C6	116.1 (3)
C7—N2—H2A	118.7	C7—C8—H8A	118.4
C10—N2—H2A	118.7	C9—C8—C7	123.2 (3)
C2—C1—H1A	109.5	C9—C8—H8A	118.4
C2—C1—H1B	109.5	C4—C9—H9A	120.3
C2—C1—H1C	109.5	C8—C9—C4	119.5 (4)
H1A—C1—H1B	109.5	C8—C9—H9A	120.3
H1A—C1—H1C	109.5	C11—C10—C15	122.0 (4)
H1B—C1—H1C	109.5	C11—C10—N2	118.9 (4)
O1—C2—H2B	110.0	C15—C10—N2	119.0 (4)
O1—C2—H2C	110.0	C10—C11—C12	120.1 (6)
C1—C2—O1	108.3 (4)	C10—C11—H11A	120.0
C1—C2—H2B	110.0	C12—C11—H11A	120.0
C1—C2—H2C	110.0	C11—C12—H12A	120.5
H2B—C2—H2C	108.4	C13—C12—C11	118.9 (5)
O1—C3—C4	114.3 (3)	C13—C12—H12A	120.5
O2—C3—O1	123.1 (4)	C12—C13—H13A	119.4
O2—C3—C4	122.6 (4)	C14—C13—C12	121.3 (5)
C5—C4—C9	118.9 (3)	C14—C13—H13A	119.4
C5—C4—C3	119.0 (3)	C13—C14—C15	120.5 (6)
C9—C4—C3	122.1 (4)	C13—C14—H14A	119.8
C4—C5—C6	121.8 (3)	C15—C14—H14A	119.8
C4—C5—H5A	119.1	C10—C15—C14	117.2 (5)
C6—C5—H5A	119.1	C10—C15—H15B	121.4
N1—C6—C7	122.4 (3)	C14—C15—H15B	121.4
			
C3—O1—C2—C1	−171.2 (4)	N1—C6—C7—N2	1.8 (6)
C2—O1—C3—O2	−4.1 (6)	C5—C6—C7—C8	1.1 (6)
C2—O1—C3—C4	178.3 (4)	N1—C6—C7—C8	−178.6 (4)
O2—C3—C4—C5	−3.9 (6)	N2—C7—C8—C9	178.7 (4)
O1—C3—C4—C5	173.7 (4)	C6—C7—C8—C9	−0.9 (6)
O2—C3—C4—C9	174.8 (4)	C7—C8—C9—C4	0.1 (6)
O1—C3—C4—C9	−7.6 (5)	C5—C4—C9—C8	0.4 (6)
C9—C4—C5—C6	−0.2 (6)	C3—C4—C9—C8	−178.4 (4)
C3—C4—C5—C6	178.6 (4)	C7—N2—C10—C11	−106.2 (5)
C4—C5—C6—N1	179.1 (4)	C7—N2—C10—C15	78.1 (5)
C4—C5—C6—C7	−0.6 (6)	C15—C10—C11—C12	−1.0 (7)
O3—N1—C6—C5	173.7 (4)	N2—C10—C11—C12	−176.6 (4)
O4—N1—C6—C5	−8.2 (6)	C10—C11—C12—C13	−0.1 (8)
O3—N1—C6—C7	−6.6 (6)	C11—C12—C13—C14	0.5 (9)
O4—N1—C6—C7	171.5 (4)	C12—C13—C14—C15	0.1 (8)
C10—N2—C7—C8	3.1 (6)	C11—C10—C15—C14	1.6 (7)
C10—N2—C7—C6	−177.4 (4)	N2—C10—C15—C14	177.2 (4)
C5—C6—C7—N2	−178.5 (4)	C13—C14—C15—C10	−1.1 (7)

### Hydrogen-bond geometry (Å, °)

**Table:**

*D*—H···*A*	*D*—H	H···*A*	*D*···*A*	*D*—H···*A*
N2—H2A···O3	0.86	1.98	2.623 (5)	131
N2—H2A···O2^i^	0.86	2.31	2.978 (4)	134

Symmetry codes: (i) *x*−1/2, −*y*−1/2, *z*−1/2.
